# Comprehensive Transcriptomic Analysis Reveals Cell-Type-Specific Roles of Human Odorant Receptors in Glioblastoma and the Tumor Microenvironment

**DOI:** 10.3390/ijms252413382

**Published:** 2024-12-13

**Authors:** Hee Jin Cho, Dong Jun Yeo, HeeWoong Yang, JaeHyung Koo

**Affiliations:** 1 Department of Biomedical Convergence Science and Technology, Advanced Institute of Science and Technology, Kyungpook National University, Daegu 41566, Republic of Korea; heejincho@knu.ac.kr (H.J.C.); iamydj0928@naver.com (D.J.Y.); 2 Cell and Matrix Research Institute, Kyungpook National University, Daegu 41944, Republic of Korea; 3 Department of New Biology, Daegu Gyeongbuk Institute of Science and Technology (DGIST), Daegu 42988, Republic of Korea; yanghw@dgist.ac.kr; 4 Korea Brain Research Institute (KBRI), Daegu 41062, Republic of Korea

**Keywords:** glioblastoma (GBM), odorant receptors (ORs), G protein-coupled receptors (GPCRs), tumor microenvironment (TME), single-cell RNA sequencing, vascular remodeling, angiogenesis, tumor-associated macrophages (TAMs), synaptic adaptation, therapeutic targets

## Abstract

Odorant receptors (ORs), which constitute approximately 50% of all human G protein-coupled receptors, are increasingly recognized for their diverse roles beyond odor perception, including functions in various pathological conditions like brain diseases and cancers. However, the roles of ORs in glioblastoma (GBM), the most aggressive primary brain tumor with a median survival of only 15 months, remain largely unexplored. Here, we performed an integrated transcriptomic analysis combining The Cancer Genome Atlas RNA-seq and single-cell RNA sequencing data from GBM patients to uncover cell-type-specific roles of ORs within the tumor and its microenvironment. Our findings reveal that ORs display distinct expression patterns, with *OR51E1* enriched in pericytes linked to vascular remodeling and angiogenesis, OR2B11 associated with tumor-associated macrophages supporting immunosuppressive phenotypes, and OR2L13 correlated with synaptic activity in recurrent tumors, potentially mediating treatment-induced neuronal adaptations. These results highlight ORs as potential therapeutic targets, offering new insights into their regulatory roles in GBM progression, immune modulation, and treatment resistance.

## 1. Introduction

Glioblastoma (GBM) remains one of the most lethal cancers, with a median survival of only 12–15 months and a recurrence rate of about 90% within two years [1,2]. The current standard treatment, which involves maximal surgical resection followed by concurrent chemoradiation with temozolomide, has shown limited effectiveness in improving patient outcomes. Despite extensive research efforts to enhance clinical outcomes, treatment resistance remains due to several key challenges. GBM exhibits remarkable inter- and intra-tumoral heterogeneity, leading to genomic and cellular evolution under therapeutic pressure [3,4,5,6]. Additionally, the plasticity of GBM’s malignant states enables tumor cells to evade treatment through state transitions [7]. The complex GBM microenvironment also plays crucial roles in disease progression and treatment resistance, characterized by hypoxia, high infiltration of tumor-associated microglia/macrophages (TAMs), and dynamic interactions with brain stromal cells [8,9,10,11]. Specifically, interactions between TAMs and glioma cells contribute to an immunosuppressive microenvironment that fosters tumor growth and facilitates resistance mechanisms [12]. These features collectively promote irregular vessel development, an immunosuppressive microenvironment with mesenchymal transition, and treatment-induced synaptic plasticity [4,8,9,13]. Furthermore, the blood–brain barrier (BBB) poses additional challenges by limiting drug delivery to tumor cells [14]. Therefore, understanding and modulating the tumor microenvironment (TME) has emerged as a key strategy to overcome current therapeutic limitations.

Precision medicine has revolutionized cancer treatment by integrating advanced diagnostic and therapeutic strategies tailored to individual patients. Among these, theranostic nanosystems, which combine diagnostic and therapeutic capabilities, have emerged as a promising approach for treating aggressive cancers. These nanosystems are particularly advantageous in neuro-oncology as they can penetrate biological barriers like the BBB due to their optimized structure and solubility characteristics. Recent studies have demonstrated that theranostic approaches enhance diagnostic precision while reducing systemic side effects through targeted delivery mechanisms [15]. The integration of omics technologies has further expanded the potential of these systems, enabling the identification of novel theranostic targets and the development of personalized treatment strategies.

In this context, odorant receptors (ORs), the largest family of G protein-coupled receptors (GPCRs), have emerged as potential theranostic targets. While best known for their role in olfactory signal transduction, emerging evidence reveals that they are widely expressed outside the olfactory system [16,17]. Their non-canonical functions have been highlighted in various diseases, including brain disorders and cancer [16,17,18,19,20,21,22,23,24]. Several studies have demonstrated their potential as therapeutic targets and prognostic biomarkers in cancer, with OR51E2, also known as the prostate-specific G-protein-coupled receptor, and OR2B6 showing overexpression in prostate and breast carcinomas, respectively [25,26]. OR51E2 overexpression has been linked to cancer cell proliferation and migration, while OR2B6 overexpression in breast carcinoma contributes to invasive behavior and metastatic potential [26,27]. In immune regulation, Olfr78, the mouse ortholog of OR51E2, partners with a GPCR to influence macrophage polarization toward a protumoral M2 phenotype [27]. Beyond cancer and immune regulation, OR dysregulation in brain stromal cells, including microglia, astrocytes, and neurons—central players in neuroinflammatory and degenerative processes—links OR function to mechanisms underlying brain disorders, highlighting their potential as therapeutic targets [21,23,28,29].

While our previous systematic analysis of adult gliomas using The Cancer Genome Atlas (TCGA) bulk RNA-seq data suggested potential therapeutic roles for ORs, the specific functions of ORs within distinct cell types of the TME remained unclear due to the limitations of bulk sequencing approaches that average signals across heterogeneous cell populations [18,19]. Given the complex cellular composition of GBM and the emerging roles of ORs in both tumor cells and the TME, a comprehensive analysis focusing on cell-type specific OR functions and their interactions with the TME is crucial. Here, we performed an integrated transcriptomic analysis by combining TCGA RNA-seq data with expanded single-cell RNA sequencing of GBM patients to identify the cell-type specific roles of ORs and their biological implications. This approach reveals previously undetected interactions between ORs and the TME that were not evident in bulk RNA-seq analyses. It also offers valuable insights into potential therapeutic strategies targeting GBM’s cellular heterogeneity and complex tumor–TME interactions.

## 2. Results

### 2.1. Comprehensive Analysis Reveals Distinct OR Expression Patterns in GBM

#### 2.1.1. Distinct OR Expression Patterns in GBM Tissues

**Figure 1:**
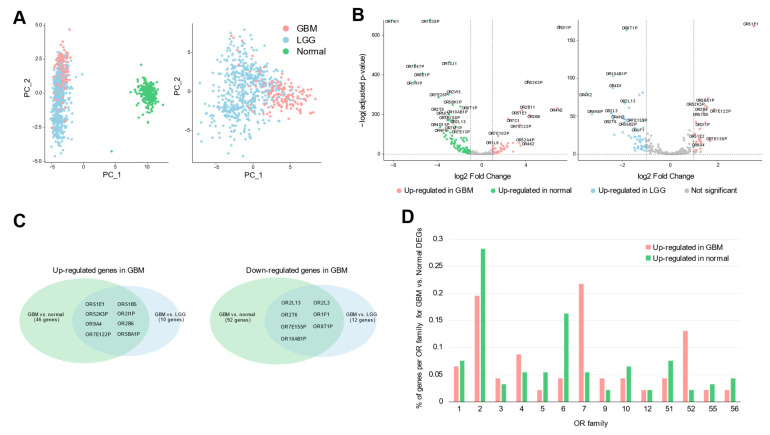
Distinct OR expression patterns in GBM compared to normal brain and LGG. (**A**) PCA of OR gene expression using TCGA and GTEx RNA-seq data. PCA was performed on the expression profiles of 847 OR genes. PCA plot including GBM, LGG (TCGA), and normal cortex (GTEx) samples (left panel). PCA plot showing the distribution of TCGA samples only (right panel). Each dot represents an individual sample, and samples from TCGA-GBM, TCGA-LGG, and GTEx normal brain are indicated in red, blue, and green, respectively. (**B**) DEG analysis of OR in GBM. Volcano plots showing differentially expressed OR genes filtered from genome-wide differential expression analysis. GBM versus normal cortex samples (left panel). GBM versus LGG samples (right panel). For clarity, only non-overlapping OR gene labels are displayed. (**C**) Venn diagrams illustrating the overlap of differentially expressed OR genes between GBM versus normal cortex and GBM versus LGG comparisons. The diagram shows OR genes that are up-regulated (left panel) and down-regulated (right panel) in GBM. (**D**) Bar plot showing the percentage of dysregulated OR genes within each OR family (calculated as the number of dysregulated OR genes divided by the total number of OR genes per family). Comparison of up- (pink) and down-regulated (green) OR percentages across 14 detected OR families.

Principal component analysis (PCA) of OR gene expression in TCGA GBM (IDH wildtype), TCGA lower grade glioma (LGG, IDH mutant), and Genotype-Tissue Expression (GTEx) cerebral cortex samples revealed distinct clustering patterns, demonstrating unique OR expression patterns in GBM compared to LGG and normal brain tissues (Figure 1A). To better understand the relationship between PCA clusters and OR expression patterns, we performed differential expression gene (DEG) analysis. The analysis revealed that the GBM-specific cluster was characterized by 46 up-regulated and 92 down-regulated ORs compared to normal brain tissue (Table S1). When compared to LGG samples, this cluster showed elevated expression of 10 ORs and decreased expression of 12 ORs. Notably, among these dysregulated ORs, eight up-regulated and seven down-regulated ORs maintained their expression patterns in both comparisons (versus normal brain and LGG), suggesting their potential involvement in GBM-specific processes (Figure 1B,C, Table S1). Visualization of OR expression levels on the PCA plot confirmed that these top up-regulated ORs showed enriched expression specifically in the GBM cluster (Figure S1). Interestingly, several of these up-regulated ORs have been previously reported in other cancers. For instance, OR2B6 overexpression has been reported in breast cancer, and *OR51E1* has been found to be elevated in advanced prostate cancer and other solid tumors, consistent with our findings [18,26,30,31,32]. The persistent dysregulation of certain ORs, such as *OR2B6* and *OR51E1*, in comparisons with both normal and LGG tissues suggests that these ORs may be involved in critical GBM-specific processes, potentially contributing to malignancy or treatment resistance. Analysis of OR family distribution revealed that OR family 7 was the most enriched (21%) in up-regulated ORs, while OR family 2 comprised the largest portion (28%) of down-regulated ORs (Figure 1D). Interestingly, a significant proportion of dysregulated ORs were pseudogenes, with 32% of up-regulated and 52% of down-regulated ORs in this category, indicating that both functional OR genes and pseudogenes may contribute to GBM pathogenesis and its microenvironment (Table S1).

#### 2.1.2. Distinct OR Expression Patterns in Neoplastic and Non-Neoplastic Cells of GBM

**Figure 2:**
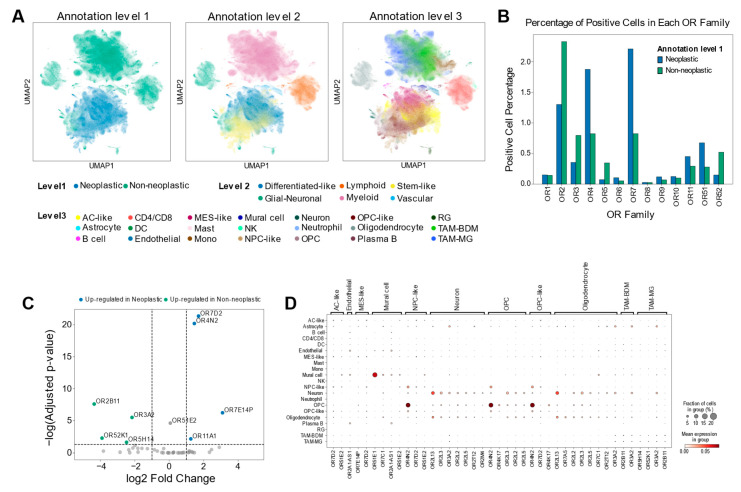
Cell-type-specific OR expression patterns in GBM at single-cell resolution. (**A**) Uniform manifold approximation and projection (UMAP) visualization of scRNA-seq data showing the distribution of cell populations in GBM. Each cell (dot) is colored by cell identity at three annotation levels (left: level 1, middle: level 2, right: level 3), following the reference publication annotation scheme [33]. (**B**) OR family distribution analysis showing the percentage of OR-positive cells across cell populations, demonstrating predominant enrichment of OR family 7 in neoplastic cells and OR family 2 in non-neoplastic cells. Cells with positive expression of any OR in each OR family were designated as OR-positive. (**C**) Differential expression analysis of OR genes between neoplastic and non-neoplastic cells. Colored dots represent differently expressed ORs (|log2 fold change| > 1, adjusted *p*-value < 0.05). (**D**) Cell-type-specific differentially expressed OR genes based on annotation level 3 (adjusted *p*-value < 0.05). Statistical significance was assessed by Wilcoxon rank-sum testing with Benjamini–Hochberg correction.

To delineate OR expression patterns at the single-cell level, we analyzed combined GBM single-cell RNA sequencing (scRNA-seq) datasets [33], comprising 1.1 million cells from 240 patients across 26 independent cohorts. The cells were hierarchically classified across three levels: first by neoplastic (tumor-derived) versus non-neoplastic (microenvironment-derived) populations (level 1), followed by major cell types (level 2), and finally by specific cell subtypes (level 3) (Figure 2A). Consistent with our TCGA GBM analysis, OR family 7 and 2 showed the highest enrichment in neoplastic and non-neoplastic cells, respectively, despite limited OR expression across cell populations (Figure 1D and Figure 2B). DEG analysis revealed that *OR4N2* was up-regulated in neoplastic cells as well as GBM tissues, suggesting its potential tumor-intrinsic function (Figure 2C and Table S1). Conversely, *OR2B11*, *OR52K1*, and *OR3A2* were up-regulated in GBM tissues and non-neoplastic cells, implying their roles in modulating the TME to promote tumorigenesis (Figure 2C and Table S1). Furthermore, cell-type-specific analysis (annotated according to the reference methodology [33]) indicated that *OR4N2* was expressed in neoplastic cells, while other genes, such as *OR2B11*, were specifically expressed in TME cells such as TAMs (Figure 2D and Table S2). The specific expression of *OR4N2* in neoplastic cells suggests a possible role in tumor cell survival or proliferation, whereas the up-regulation of *OR2B11* in TAMs points to a role in shaping the TME to support tumor growth. The cell-type specificity of OR expression was further validated through correlation analysis between OR expression levels and cell-type-specific gene set scores in TCGA GBM tissues (Figure S2).

### 2.2. Heterogeneous OR Expression Across Different GBM Malignant States

**Figure 3:**
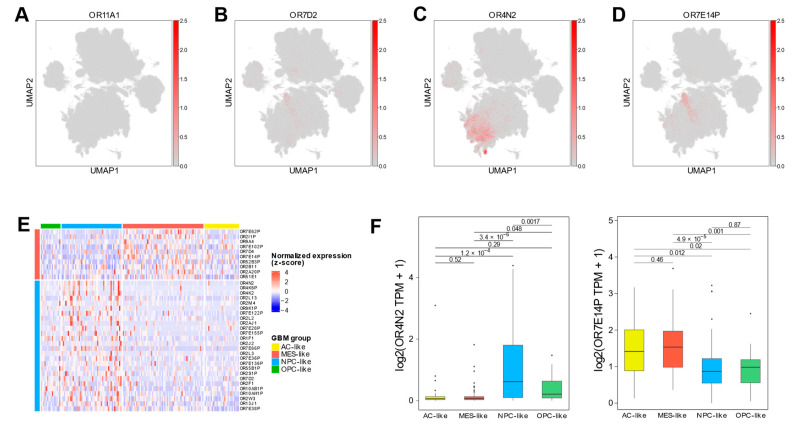
Neoplastic cell-expressing OR genes in GBM. (**A**–**D**) UMAP visualization of scRNA-seq data showing OR expression distribution across neoplastic and non-neoplastic cells. The analysis demonstrates expression patterns of *OR11A1* (**A**), *OR7D2* (**B**), *OR4N2* (**C**), and *OR7E14P* (**D**). (**E**) Heatmap depicting state-specific OR gene expression in TCGA-GBM data. DEG analysis identified up-regulated OR genes in each GBM state-enriched group (OPC-, NPC-, MES-, astrocyte (AC)-like) compared to other groups. The analysis reveals OR genes were specifically up-regulated in MES- and NPC-like groups, while no significant OR genes were detected in OPC- and AC-like groups. (**F**) Box plots showing differential expression of *OR4N2* (left panel) and *OR7E14P* (right panel) across GBM state-enriched groups, with statistical significance determined by Student’s *t*-test.

To examine the heterogeneity of OR expression within tumor cells, we analyzed OR expression patterns across four distinct GBM neoplastic cell states. Among the neoplastic-up-regulated OR genes, *OR11A1* showed uniformly weak expression, while *OR7D2* exhibited significant expression across all malignant states (Figure 3A,B, Table S2). In contrast, *OR4N2* and *OR7E14P* demonstrated state-specific patterns, with predominantly enriched in neural precursor cell (NPC)/oligodendrocyte precursor cell (OPC)-like and mesenchymal (MES)-like states, respectively (Figure 3C,D). These findings highlighted the distinct regulation of ORs in different GBM cellular states. Despite minor discrepancies due to cellular plasticity and intra-tumoral heterogeneity, where multiple malignant states coexist within bulk tissue samples [7], validation using TCGA GBM bulk tissue data corroborated these patterns. *OR4N2* exhibited significantly higher expression in tumors with NPC-like features, while *OR7E14P* showed elevated expression in tumors with MES-like characteristics compared to other tumors (Figure 3E,F).

Given emerging evidence suggesting that pseudogenes can function as regulatory elements in cancer, we performed weighted correlation network analysis (WGCNA) using TCGA-GBM data (Figure S3). This analysis identified 27 distinct modules, with OR7E14P showing significant correlation with the pink module (gene significance = 0.61, *p* = 1 × 10^17^ (Figure S3C). Within this module, we identified 16 genes that were highly connected to OR7E14P. Gene ontology analysis of these co-expressed genes revealed significant enrichment in cytoskeleton-related pathways, particularly in cilium and microtubule bundle formation (Figure S3D,E). These findings are particularly relevant as cytoskeletal reorganization is a key feature of proneural to mesenchymal transition in GBM [34], consistent with our cell type-specific analysis showing OR7E14P enrichment in the MES-like state.

Although these state-associated OR genes showed no significant association with clinical outcomes (Figure S4), these distinct expression patterns suggest that ORs may play specific roles in the unique biology of each malignant state, potentially influencing pathways linked to tumor plasticity and adaptive behaviors.

### 2.3. Pericyte-Enriched OR51E1 Promotes Vascular Remodeling and Angiogenesis in GBM

**Figure 4:**
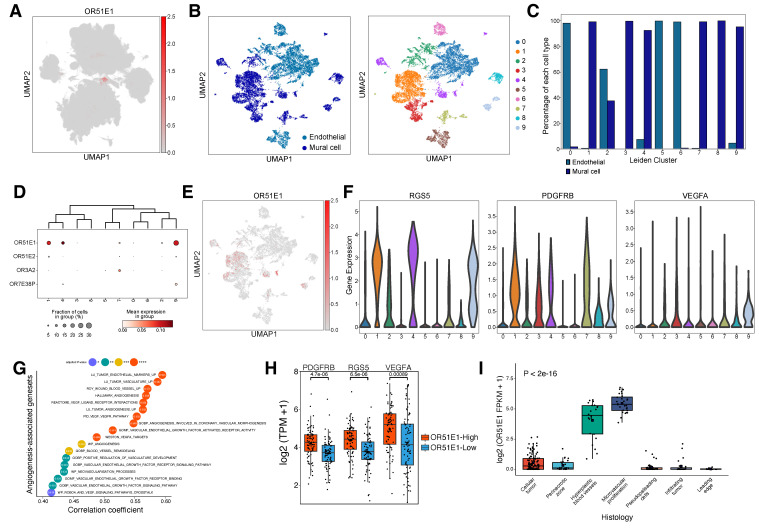
*OR51E1* contributes to vascular remodeling and angiogenesis in GBM. (**A**) UMAP visualization of scRNA-seq data showing the *OR51E1* expression distribution across neoplastic and non-neoplastic cells, with notable expression in vascular regions. (**B**) Sub-clustering analysis of 11,974 vascular cells. Each cell (dot) is colored by cell type (left panel) and assigned Leiden cluster (right panel). (**C**) Bar plot showing the proportion of each cell type within individual Leiden clusters. (**D**) DEG analysis of OR genes across Leiden clusters (adjusted *p*-value < 0.05). (**E**) UMAP visualization demonstrating *OR51E1* expression distribution across vascular cells, with prominent expression in clusters 1, 4, and 9. (**F**) Violin plots showing expression levels of pericyte markers and angiogenic factors: *RGS5* (left), *PDGFRB* (middle), and *VEGFA* (right panel). (**G**) Dot plot showing the results of correlation analysis between *OR51E1* expression and angiogenesis-associated gene sets from the Molecular Signatures Database (MSigDB) in TCGA-GBM. **** *p* < 0.0001; *** *p* < 0.001; ** *p* < 0.01; * *p* < 0.05. (**H**) Box plots showing differential expression of *PDGFRB*, *RGS5*, and *VEGFA* between *OR51E1*-high (>median) and -low (<median) groups in TCGA-GBM data. (**I**) Box plot showing *OR51E1* expression across distinct histological features from the Ivy Glioblastoma Atlas dataset, including cellular tumor, perinecrotic zone, hyperplastic blood vessels, microvascular proliferation, pseudopalisading cells, infiltrating tumor, and leading edge. Statistical significance was assessed by ANOVA.

Cell-type-specific OR expression analysis identified vascular-associated OR genes, including *OR51E1*, in endothelial cells and pericytes (mural cells) (Figure 2D and Figure 4A). Sub-clustering analysis of vascular cells using scRNA-seq data revealed 10 distinct clusters (Figure 4B), with clear separation between mural and endothelial cell populations (Figure 4C). Clusters 0, 5, and 6 were enriched for endothelial cells, while clusters 1, 3, 4, 7, 8, and 9 were dominated by mural cells, with cluster 2 showing mixed populations. Among vascular-associated ORs, *OR51E1*, *OR51E2*, *OR3A2*, and *OR7E38P* demonstrated cluster-specific expression patterns (Figure 4D,E, Table S3). Notably, *OR51E1* exhibited prominent expression in clusters 1, 4, and 9, which were characterized by elevated levels of pericyte markers *RGS5* and *PDGFRB* (Figure 4F). VEGFA, a key regulator of vascular remodeling and angiogenesis [35], was predominantly expressed in GBM neoplastic cells rather than vascular cells (Figure S5), suggesting potential paracrine signaling between VEGFA-secreting tumor cells and *OR51E1*-positive pericytes. Analysis of the TCGA GBM dataset revealed *OR51E1*’s strong correlations with vascular cell populations as well; correlation analysis with cell type-specific gene sets demonstrated significant positive associations with both mural (r = 0.63) and endothelial cells (r = 0.4), with mural cells showing stronger correlation (Figure S6). Consistently, *OR51E1* expression showed significant positive correlations with gene sets of blood vessel remodeling and angiogenesis (Figure 4G). Furthermore, *OR51E1*-high GBM tissues showed significantly elevated expression of *RGS5*, *PDGFRB*, and *VEGFA* compared to *OR51E1*-low samples (Figure 4H). The anatomical analysis using the Ivy Glioblastoma Atlas dataset further substantiated these findings, demonstrating significant *OR51E1* up-regulation in regions of microvascular proliferation and hyperplastic blood vessels (Figure 4I). Taken together, these results suggest that *OR51E1* plays a crucial role in GBM vasculature, particularly in pericyte-mediated vascular remodeling and tumor angiogenesis, potentially influencing tumor progression and therapeutic resistance.

### 2.4. OR Genes Modulate GBM Aggressiveness via TAM-Mediated Mechanisms

**Figure 5:**
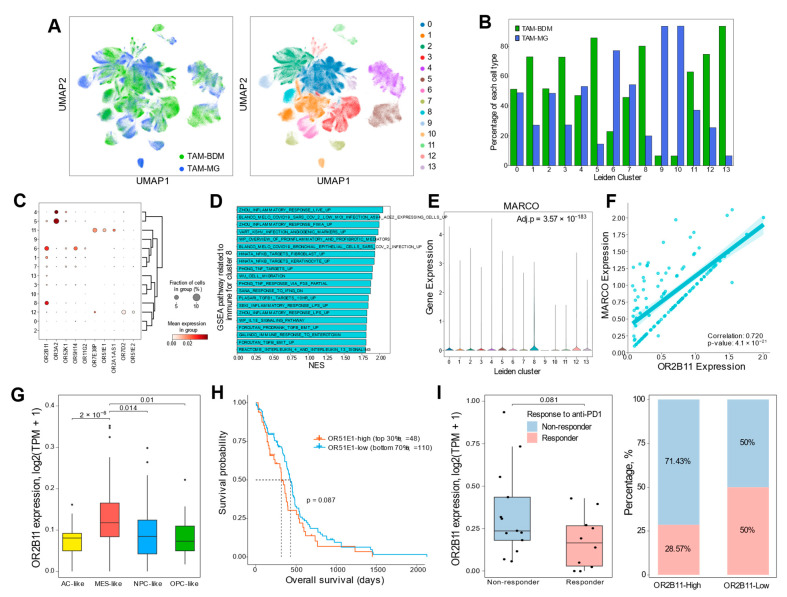
OR2B11 overlaps with mesenchymal shift-promoting TAM population in GBM. (**A**) Sub-clustering analysis of 468,339 TAM cells. Each cell (dot) is colored by cell type (left panel) and assigned Leiden cluster (right panel). (**B**) Bar plot showing the proportion of each cell type within individual Leiden clusters. (**C**) DEG analysis of OR genes across Leiden clusters (adjusted *p*-value < 0.05). (**D**) Bar plot showing gene set enrichment analysis (GSEA) of immune-related signatures in cluster 8. NES, normalized enrichment score. (**E**) Violin plot showing *MARCO* expression levels across Leiden clusters. Statistical significance between cluster 8 and other clusters was assessed by Wilcoxon rank-sum testing with Benjamini–Hochberg correction. (**F**) Scatter plot showing correlation between *OR2B11* and *MARCO* expression, using only cells expressing both genes in cluster 8. The solid line and shadow indicate the linear regression trend with 95% confidence intervals. (**G**) Box plot showing differential expression of *OR2B11* in the MES-like-enriched group compared to other groups, with statistical significance assessed by Student’s *t*-test. (**H**) Kaplan–Meier survival analysis of TCGA-GBM cohort comparing *OR2B11*-high (top 30%) and *OR2B11*-low (bottom 70%) groups. Statistical significance was assessed by log-rank test. (**I**) Analysis of *OR2B11* expression in relation to anti-PD1 treatment response. Box plot showing *OR2B11* expression levels between response groups, with statistical significance assessed by Student’s *t*-test (left panel). Bar plot showing the proportion of responders and non-responders stratified by *OR2B11* expression levels, with high and low groups defined by top 30% threshold (right panel).

We identified *OR2B11*, *OR3A2*, and *OR52K1* as GBM TME-specific genes with enriched expression in TAMs (Figure 1 and Figure 2). To understand their roles in TAM populations, including bone marrow-derived macrophages (TAM-BDM) and microglia (TAM-MG), we performed sub-clustering analysis using scRNA-seq data, revealing 14 distinct clusters (Figure 5A). The TAM sub-clusters showed heterogeneous distribution of TAM-BDM and TAM-MG populations, with distinct OR expression patterns (Figure 5B,C, Table S4). Among these OR genes, *OR2B11* showed robust positive correlation with both TAM-BDM and TAM-MG in TCGA GBM tissues, consistent with scRNA-seq analysis (Figures S2A and S7). Notably, cluster 8, enriched in TAM-BDM and characterized by elevated NF-κB and TGF-β signaling, showed exclusive *OR2B11* expression among OR genes (Figure 5C and D). These pathways are key regulators of mesenchymal transition in GBM [13,36,37]. Previous studies have demonstrated that MARCO-high TAMs promote immunosuppressive TME and aggressive tumor phenotypes by enhancing radiation resistance, stemness, and mesenchymal transition [9,38]. *MARCO* was up-regulated in cluster 8, and *OR2B11* showed significant positive correlation with *MARCO* expression in this cluster (Figure 5E,F). Consistently, *OR2B11* exhibited significantly higher expression in the mesenchymal subtype compared to other subtypes (Figure 5G). Although the statistical significance was modest, *OR2B11* expression tended to be associated with poor survival (*p* = 0.087) and reduced response to anti-PD1 immunotherapy (*p* = 0.081) (Figure 5H,I). Collectively, these results suggest that OR2B11 may serve as a potential therapeutic target for GBM by promoting mesenchymal transition and immunosuppressive TME through TAM regulation, potentially driving aggressive tumor behavior.

### 2.5. OR2L13 Reflects Synaptic Adaptations in Recurrent GBM Following Treatment

**Figure 6:**
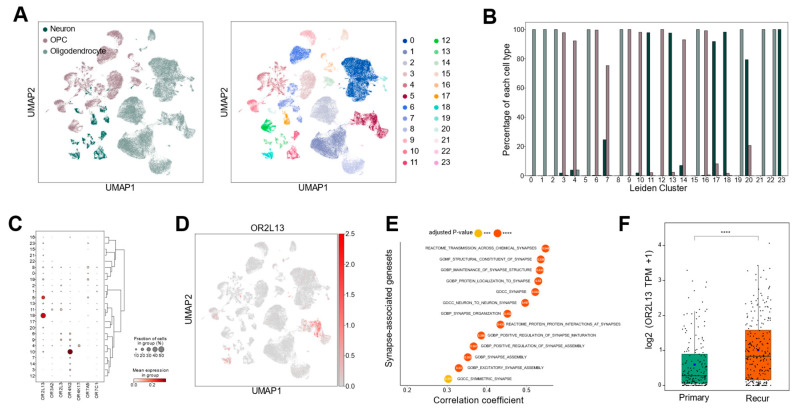
OR2L13, expressed in oligodendrocytes and neurons, contributes to treatment-induced synaptic plasticity in recurrent GBM. (**A**) Sub-clustering analysis of 81,600 neurons, OPCs, and oligodendrocytes. Each cell (dot) is colored by cell type (left panel) and assigned Leiden cluster (right panel). (**B**) Bar plot showing the proportion of each cell type within individual Leiden clusters. (**C**) DEG analysis of OR genes across Leiden clusters (adjusted *p*-value < 0.05). (**D**) UMAP visualization demonstrating *OR2L13* expression distribution across neurons, oligodendrocytes, and OPCs, with prominent expression in subsets of neurons and oligodendrocytes. (**E**) Dot plot showing the results of correlation analysis between *OR2L13* expression and synapse-associated gene sets from MSigDB in TCGA-GBM. **** *p* < 0.0001; *** *p* < 0.001. (**F**) Box plot showing differential expression of *OR2L13* between primary and recurrent samples from Glioma Longitudinal AnalySiS (GLASS) dataset. Statistical significance was assessed by Student’s *t*-test (**** *p* < 0.0001).

*OR2L13* was identified as a significantly down-regulated OR gene in GBM compared to normal and LGG tissues (Figure 1B and Table S1), with cell-type-specific expression analysis revealing its predominant expression in oligodendrocytes and neurons rather than tumor cells (Figure 2D, Figures S1B and S8). To further characterize its expression pattern, we performed sub-clustering analysis of neurons, oligodendrocytes, and OPCs using scRNA-seq data (Figure 6A). *OR2L13* was primarily expressed in clusters enriched for oligodendrocytes and neurons, with minimal expression in OPCs (Figure 6B–D, Table S5). Oligodendrocytes and neurons orchestrate synapse activation through myelination-dependent regulation of action potential propagation and neurotransmitter release at synaptic junctions [39]. Notably, a recent study demonstrated that recurrent GBMs after treatment exhibited enhanced synaptic activity compared to their pre-treatment counterparts [4]. Analysis of the TCGA GBM dataset revealed significant positive correlations between *OR2L13* expression and synapse-associated gene sets (Figure 6E). Consistently, *OR2L13* showed significant up-regulation in recurrent tumors in the Glioma Longitudinal AnalySiS (GLASS) dataset (Figure 6F). These findings suggest that OR2L13 may play a role in treatment-driven neuronal adaptation within GBM, potentially influencing synaptic pathways that support tumor recurrence and therapy resistance.

## 3. Discussion

In this study, we conducted comprehensive analysis to elucidate cell-type-specific expression and functions of ORs in GBM. Specifically, we identified distinct roles of ORs in the complex cellular composition and dynamic interactions between GBM tumor cells and TME, suggesting ORs as potential therapeutic targets.

Our analysis first revealed distinctive OR expression patterns in tumor cells, particularly highlighting the predominant role of OR family 7. The enrichment of OR family 7 in GBM is particularly noteworthy, as a member of this family, OR7E156P, was previously reported to promote tumor growth and invasion through the OR7E156P/miR-143/HIF1A axis [40]. This suggests that OR families may retain evolutionarily conserved features that can be reactivated during GBM development. Furthermore, we identified two distinct patterns of OR expression in malignant cells: *OR7D2* showed uniformly elevated expression across all malignant cellular states compared to non-neoplastic cells, suggesting its fundamental role in GBM progression, while *OR4N2* and *OR7E14P* exhibited state-dependent expression patterns, indicating their involvement in regulating GBM cellular plasticity.

Intriguingly, we found widespread expression of OR pseudogenes in GBM. Although this observation could partly stem from technical limitations due to high sequence homology among OR genes, the experimentally validated functional role of OR7E156P in transcriptional regulation suggests broader involvement of OR pseudogenes in GBM progression [40]. These findings expand our understanding of OR-mediated gene regulation in GBM beyond canonical protein-coding genes and highlight the potential importance of OR pseudogenes as regulatory factors. Pseudogenes can influence tumor biology through various mechanisms. For instance, OR7E14P can form chimeric transcripts within neighboring genes like PLEKHA7 [41], potentially altering pathways related to cellular adhesion and invasion. Second, pseudogene expression has been linked to transcriptional regulation, potentially modulating the activity of adjacent functional OR genes. This mechanism, previously proposed in olfactory sensory neurons [42], may similarly influence tumor-related gene networks in GBM. Additionally, pseudogene-derived RNAs may act as competing endogenous RNAs, sequestering miRNAs [43] and thereby modulating oncogenic or tumor-suppressive pathways. Our bulk and single-cell RNA-seq analysis revealed significant pseudogene expression in both neoplastic (e.g., OR7E14P) and TME-associated cells (e.g., OR7E38P), highlighting their potential regulatory roles. Notably, WGCNA showed OR7E14P correlating with cytoskeleton-related gene networks (Figure S3), suggesting a role in promoting mesenchymal transition and invasive plasticity through enhanced cytoskeletal reorganization [34]. Overall, these results suggest that pseudogenes may actively contribute to the regulatory networks involved in GBM progression and shaping the TME, rather than being merely non-functional genomic elements. Future studies should further investigate the broader roles of OR pseudogenes as therapeutic targets or biomarkers in GBM.

Our analysis revealed three distinct OR-mediated regulatory mechanisms in the TME that could have therapeutic implications. First, *OR51E1* exhibited pericyte-specific enrichment and demonstrated a significant association with vascular remodeling and angiogenesis, particularly in regions of microvascular proliferation. While *OR51E1* overexpression has been previously reported in various solid tumors [18,30,31,32], our study elucidates its specific role in vascular regulation through pericyte-mediated mechanisms. This finding aligns with previous research showing that *OR51E1*, and its murine ortholog Olfr558, is expressed in renin-positive cells in the kidney and in vascular smooth muscle cells, where it modulates blood pressure by influencing renin expression and vascular reactivity [44,45]. Given its established involvement in vascular remodeling and hemodynamics, *OR51E1* may have a broader physiological role, influencing vascular function. Considering the limited efficacy of current anti-angiogenic monotherapies in GBM and other solid tumors, *OR51E1* presents a promising complementary therapeutic target for modulating tumor vasculature and potentially enhancing therapeutic outcomes.

Second, *OR2B11* demonstrated a strong association with immunosuppressive TAM phenotypes and mesenchymal GBM, which is characterized by extensive TAM infiltration and poor clinical outcomes. Notably, co-expression of *OR2B11* with *MARCO* and its correlation with NF-κB/TGF-β signaling pathways, known mediators of mesenchymal transition and radio-resistance, indicates its potential role in orchestrating an immunosuppressive TME [9,13,37,38]. This is further supported by the higher expression of OR2B11 in non-responders to anti-PD1 treatment [46], despite marginal statistical significance. Together with previous findings of Olfr78-mediated M2 phenotype generation [27], these findings suggest that ORs function as critical regulators of innate immune functions in GBM TME and represent promising targets for enhancing immunotherapeutic efficacy.

Third, *OR2L13* exhibited positive correlation with differentiated oligodendrocytes (distinct from OPCs), neurons, and synaptic assembly gene sets, with notable up-regulation in recurrent tumors. This expression pattern provides novel insights into treatment-induced neuronal transition and GBM recurrence mechanisms [4], suggesting that OR2L13-mediated synaptic regulation could be crucial for preventing tumor recurrence. Furthermore, understanding this mechanism could have broader implications for therapeutic strategies in other neurological disorders where synaptic dysregulation plays a key role.

As GPCRs, ORs present both opportunities and challenges in the context of therapeutic development. While their potential as druggable targets is promising, the identification of specific ligands and understanding of their signaling mechanisms require further investigation. Interestingly, OR gene expression in normal brain cells suggests their potential role in maintaining normal cellular functions. While our study primarily focused on pro-tumoral effects of ORs in GBM, their expression in normal brain cells raises the promising possibility that enhancing OR-mediated normal brain cell functions could contribute to anti-tumoral effects, representing an alternative therapeutic strategy.

Using bulk and scRNA-seq analysis, recent studies have provided additional insights into the roles of GPCRs in GBM [47,48]. Guo et al. demonstrated that the combination of GPCR expression and TME classification can serve as an effective prognostic marker for GBM [47]. While their findings align with our observations regarding the importance of GPCRs in the TME, our study provides complementary insights by specifically focusing on the OR family. In particular, we identified distinct roles of ORs in GBM cellular states and TME regulation, extending the current understanding of GPCR-mediated mechanisms in GBM. Based on these findings, future studies could explore the development of machine learning-based classification models using OR expression patterns, which might provide new opportunities for patient stratification and personalized therapeutic approaches.

In this study, we performed comprehensive transcriptomic analysis integrating bulk RNA-seq and scRNA-seq data. However, several limitations need to be addressed in future studies. First, experimental validation through ligand identification, signaling mechanism characterization, and in vivo functional studies are essential. Second, the therapeutic potential of targeting ORs needs to be rigorously evaluated, particularly considering the challenge of BBB permeability in GBM treatment. Third, the clinical implications of our findings require validation in larger patient cohorts.

While our study focuses on GBM due to its unique biological context—the brain’s specialized environment—we acknowledge the importance of a pan-cancer analysis to identify ORs consistently dysregulated across cancer types. Based on this study, the functions of ORs using pan-cancer bulk and scRNA-seq datasets warrant further investigation.

Future studies integrating transcriptomic data, histolomics, and radiomics with OR-based approaches could provide more comprehensive insights into GBM biology and treatment. Such analysis with multi-layer data could enhance patient stratification and treatment selection with artificial intelligence approaches. This integrated approach, combining molecular and imaging data with OR-targeted therapies, represents a promising direction for improving both diagnostic accuracy and therapeutic efficacy. Nevertheless, our study establishes a foundation for understanding the diverse roles of ORs in GBM pathobiology and presents promising opportunities for therapeutic development.

## 4. Materials and Methods

### 4.1. Collection of Data

Bulk RNA-seq data for this study were obtained from various sources. Low-Grade Glioma (LGG) [49] and Glioblastoma Multiforme (GBM) [3,50] datasets from The Cancer Genome Atlas (TCGA) comprising raw count, transcripts per million (TPM), and fragments per kilobase million (FPKM) values were downloaded with ‘GDCquery’ function included in R package ‘TGGAbiolinks’ [51]. For the TCGA-GBM dataset, IDH mutant samples were filtered out, and only 159 IDH wildtype samples were used in this study. Additional glioma datasets were acquired from the Glioma Longitudinal AnalySiS (GLASS) (https://www.synapse.org/Synapse:syn26465623, accessed on 15 October 2024) [6,52] and the Ivy Glioblastoma Atlas Project (https://glioblastoma.alleninstitute.org/static/download.html, accessed on 15 October 2024) [53], including raw count, TPM values, and FPKM values, respectively. Cerebral cortex data for normal brain consisting of raw count and TPM values were obtained from the Genotype-Tissue Expression (GTEx) project (https://gtexportal.org/home/downloads/adult-gtex/bulk_tissue_expression, accessed on 25 October 2024) [54]. RNA-Seq data of GBM samples by anti-PD1 treatment were retrieved from the SRA under the project PRJNA482620 (https://www.ncbi.nlm.nih.gov/bioproject/PRJNA482620, accessed on 26 July 2022) [46]. Raw data were downloaded and mapped to the hg19 reference genome using ‘STAR’ (version 2.7.9a) [55]. TPM values were calculated using the ‘TPMCalculator’ (version 0.0.3) [56].

For GBM single-cell RNA sequencing data, the extended GBmap data was acquired from cellxgene (https://cellxgene.cziscience.com/collections/999f2a15-3d7e-440b-96ae-2c806799c08c, accessed on 19 September 2024) [33].

### 4.2. Single-Cell RNA Sequencing Data Processing

The scRNA-seq data were analyzed using the ‘Scanpy’ (version 1.10.2, Theis lab, Munich, Germany) Python library [57]. Log-normalized counts were used for further analysis. Cell-type-specific gene expression was identified by ‘scanpy.tl.rank_genes_groups’ with the Willcoxon rank-sum method and Benjamini–Hochberg correction method [58]. For each clustering analysis, highly variable genes were identified by ‘scanpy.pp.highly_variable_genes’ with default parameters (min_disp = 0.5, max_disp = inf, min_mean = 0.0125, max_mean = 3, span = 0.3, n_bins = 20, flavor = ’seurat’). Dimensionality reduction using principal component analysis (PCA) was performed using ‘scanpy.pp.pca’ with only highly variable genes and default parameters (zero_center = True, svd_solver = arpack, use_highly_variable = True). The top 30 principal components were used for downstream analyses. The nearest neighbors distance matrix was computed by ‘scanpy.pp.neighbors’ with n_neighbors set to 15. For visualizing the data, uniform manifold approximation and projection (UMAP) was computed using ‘scanpy.tl.umap’ with default parameters. Cell clustering was performed with the Leiden algorithm using ‘scanpy.tl.leiden’ with a resolution of 0.1 and default parameters to identify distinct cell populations.

### 4.3. Pathway Enrichment Analysis for scRNA-seq

Pathway enrichment analysis was conducted as described in Heumos et al., 2023 [59]. Briefly, the fast gene set enrichment analysis (FGSEA) algorithm [60] implemented in ‘decoupleR’ (version 1.8.0, Saez-Rodriguez lab, Heidelberg, Germany) Python library [61] with the C2 collection of the Molecular Signatures Database (MSigDB) (version 7.5.1) [62] was used for cluster-specific pathway enrichment analysis. The function ‘decoupler.run_gsea’ was used for GSEA with 10,000 permutations and default parameters. For false discovery rate control, zero *p*-values from GSEA results were substituted with 0.0001 and corrected by the ‘scipy.stats.false_discovery_control’ function in the ‘SciPy’ (version 1.14.1) Python library [63]. Additionally, the AUCell algorithm [64] was used to score pathway activity at the single-cell level, using ‘decoupler.run_aucell’ with default parameters. Correlation between cell type-specific OR gene expression and AUCell scores was calculated using Pearson correlation with ‘scipy.stats.pearsonr’.

### 4.4. Differential Expression Gene Analysis

Differential expression gene (DEG) analysis was performed using the R package ‘DESeq2’ [65] and visualized with R package ‘ggplot2’ [66]. GBM, LGG, and normal brain RNA-Seq data from TCGA and GTEx were utilized to conduct comparison between GBM and LGG, as well as GBM and normal tissue. Through DEG analysis, up-regulated and down-regulated OR genes were identified specific to GBM. Up-regulation and down-regulation were defined based on an absolute value of log2 fold change higher than 1.0 and a *p*-value adjusted by the Bonferroni method less than 0.05. OR genes that did not fulfill these criteria were considered as not significant genes.

### 4.5. Correlation Analysis with Gene Set Scores

Correlation analysis was conducted using R package ‘Hmisc’ with the Pearson method. *p*-values from the analysis were adjusted by the Bonferroni method. In analysis between OR expression and the gene set of pathways, gene sets were acquired from the C2, C5, and Hallmark collections of MSigDB (version 7.5.1) [62]. The single-sample gene set enrichment analysis (ssGSEA) scores were calculated for each TCGA-GBM tumor sample using the ‘GSVA’ function from the ‘GSVA’ package for the given gene sets [67].

In analysis between OR expression and the gene set of cell types, gene sets for each cell type were obtained from the top 100 genes from the results of single cell RNA-sequencing. Analysis was performed using the same approach described above. The results were visualized using R package ‘ggplot2’ [66] and ‘ggpubr’ [68].

### 4.6. Survival Analysis

Survival analysis was performed using RNA-Seq and clinical data from TCGA-GBM. The Kaplan–Meier survival analysis was conducted with R package ‘Survival’ [69], and the survival curve was visualized with the ggsurvplot function from R package ‘survminer’ [70]. GBM tumor samples were categorized into high and low groups based on the expression level of OR genes. The top 30% of samples by OR gene expression was classified as the high group, and the remaining samples were designated as the low group. Survival analysis was then performed to compare the prognosis between high and low groups.

### 4.7. GBM Subtyping

Subtyping of GBM tumor samples was conducted by using TCGA GBM RNA-Seq data and the ‘GSVA’ R package [67]. Gene sets defining the mesenchymal, classical, proneural, and neural GBM subtypes were acquired from Verhaak et al. [71]. The ssGSEA scores were calculated with the gene sets, and z-scores were computed using the ‘scale’ function in R. The subtype with the highest z-score among these four subtypes was designated as the primary subtype for each GBM tumor sample.

For AC-like, MES-like, OPC-like, and NPC-like subtypes, gene sets were defined using the top 100 differentially expressed genes confirmed though single-cell RNA sequencing data. Subtyping of each sample was performed using the same ssGSEA and z-score approach described above.

### 4.8. Weighted Correlation Network Analysis (WGCNA)

We performed WGCNA using the R package ‘WGCNA’ [72]. Only genes expressed in over 95% of IDH-wildtype TCGA-GBM samples were included in the analysis. Using signed connectivity analysis with minModuleSize = 100, we identified 27 distinct modules. For the trait analysis, samples were categorized as *OR7E14P*-high (top 30%, coded as 1) or *OR7E14P*-low (bottom 30%, coded as 0). The adjacency analysis identified 16 genes highly connected to *OR7E14P*, and the co-expression network was visualized using the graph.adjacency function.

### 4.9. Gene Ontology Analysis

Gene ontology analysis was performed on *OR7E14P* and its 16 neighbor genes using the enrichGO function from the clusterProfiler R package [73]. *p*-values were adjusted using the Bonferroni method, with an adjusted *p*-value cutoff of 0.05.

### 4.10. Statistical Analysis

Statistical analysis was performed with R version 4.1.2. A Student’s *t*-test was utilized in analyzing data. TPM and FPKM values were log2 transformed for statistical analysis.

## 5. Conclusions

In this study, we comprehensively analyzed OR expression patterns in GBM using bulk and single-cell RNA sequencing, uncovering diverse roles of ORs in tumor heterogeneity, malignancy, and modulation of the tumor microenvironment. Our findings suggest that ORs could serve as therapeutic targets, given their impact on cellular plasticity, treatment resistance, and key processes within GBM. These insights provide a strong foundation for future research into OR-targeted therapies and the roles of ORs in GBM pathogenesis.

## Data Availability

The public datasets analyzed in this study are available from the following sources: 1) The Cancer Genome Atlas (TCGA) data for LGG and GBM are accessible through the GDC Data Portal. The Glioma Longitudinal AnalySiS (GLASS) consortium data are available at Synapse (syn26465623, https://www.synapse.org/Synapse:syn26465623, accessed on 15 October 2024). The Ivy Glioblastoma Atlas Project data can be accessed through https://glioblastoma.alleninstitute.org/static/download.html, accessed on 15 October 2024. Normal brain tissue expression data were obtained from the Genotype-Tissue Expression (GTEx) project portal (https://gtexportal.org/home/downloads/adult-gtex/bulk_tissue_expression, accessed on 15 October 2024). RNA-Seq data for anti-PD1 treated GBM samples are available in the Sequence Read Archive (SRA) under BioProject accession number PRJNA482620. Single-cell RNA sequencing data from the extended GBmap are accessible through cellxgene (https://cellxgene.cziscience.com/collections/999f2a15-3d7e-440b-96ae-2c806799c08c, accessed on 15 October 2024).

## Supplementary Materials

The following supporting information can be downloaded at: https://www.mdpi.com/article/10.3390/ijms252413382/s1.

## Abbreviations

OR(s)	Odorant receptor(s)
GBM	Glioblastoma
TCGA	The Cancer Genome Altas
scRNA-seq	Single-cell RNA sequencing
TME	Tumor microenvironment
TAM(s)	Tumor-associated macrophage(s)
BBB	Blood–brain barrier
LGG	Lower-grade glioma
IDH	Isocitrate dehydrogenase
GPCR(s)	G protein-coupled receptor(s)
TAM-BDM	Bone marrow-derived macrophages (type of TAM)
TAM-MG	Microglia (type of TAM)
NPC(s)	Neural precursor cell(s)
OPC(s)	Oligodendrocyte precursor cell(s)
MES	Mesenchymal
PCA	Principal component analysis
DEG	Differential expression gene
UMAP	Uniform Manifold Approximation and Projection
WGCNA	Weighted correlation network analysis
NPC	Neural progenitor cell
AC	Astrocyte-like
GLASS	Glioma Longitudinal AnalySiS
GTEx	Genotype-Tissue Expression
GSEA	Gene set enrichment analysis
ssGSEA	Single-sample gene set enrichment analysis
MSigDB	Molecular Signatures Database
FGSEA	Fast gene set enrichment analysis
TPM	Transcripts per million
FPKM	Fragments per kilobase million
