# Supplementary material for: Comprehensive Transcriptomic Analysis Reveals Cell-Type-Specific Roles of Human Odorant Receptors in Glioblastoma and the Tumor Microenvironment
Source: Int J Mol Sci. 2024 Dec 13;25(24):13382. doi: 10.3390/ijms252413382 (PMC11676228; doi:10.3390/ijms252413382)
Supplement: Supplementary file 1 [file ijms-25-13382-s001.zip › Revised supplementary materials_Tract_Yeo et al_ijms-3337550.docx]

**Supplementary Materials**


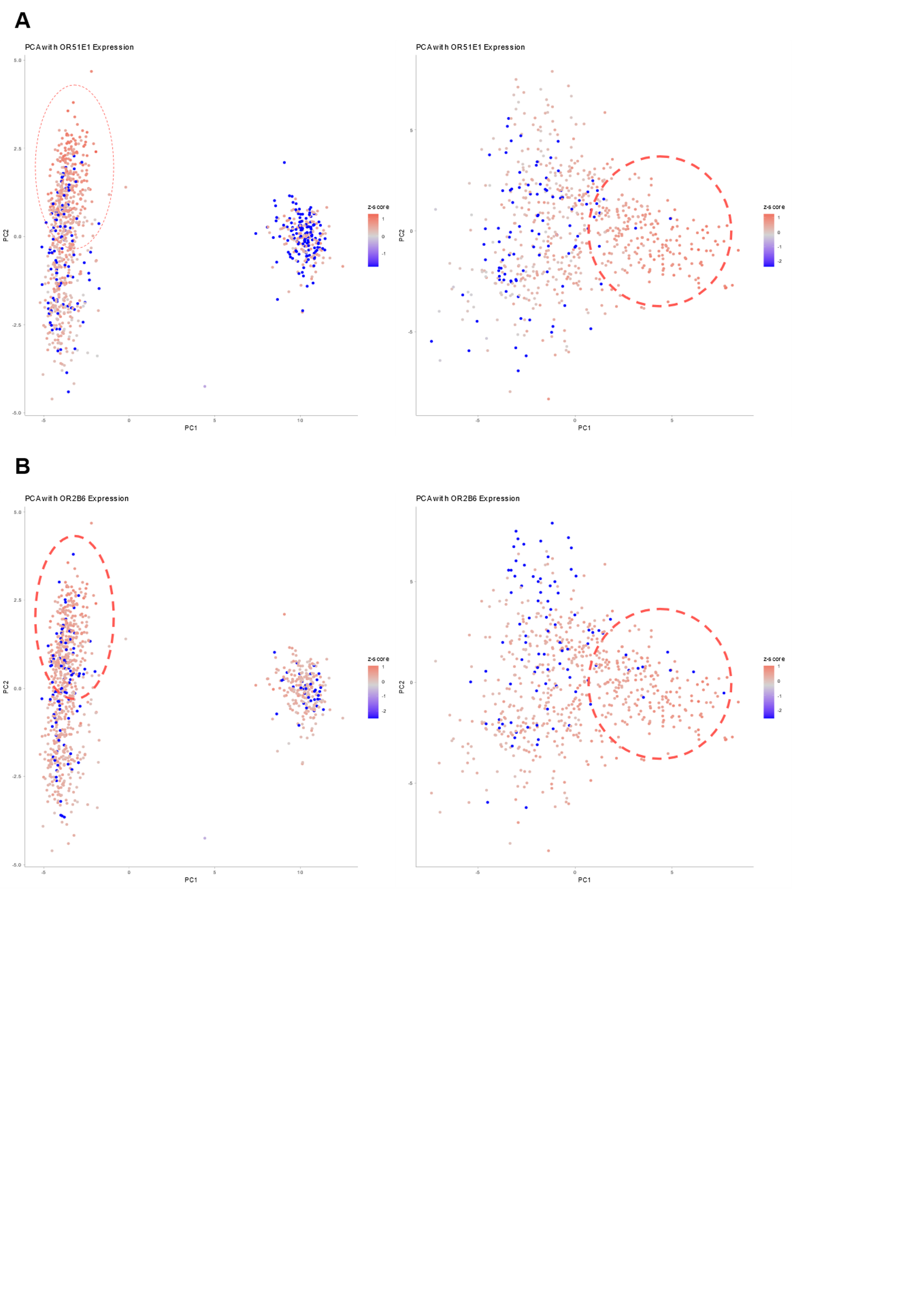


**Figure S1**. PCA of OR gene expression using TCGA and GTEx RNA-seq data. (**A-B**) PCA plot including GBM, LGG (TCGA), and normal cortex (GTEx) samples (left panel). PCA plot showing the distribution of TCGA samples only (right panel). The color scale indicates the normalized expression levels of *OR51E1* (**A**) and *OR2B6* (**B**). The red dashed circle line highlights the GBM cluster, as described in Figure 1A.


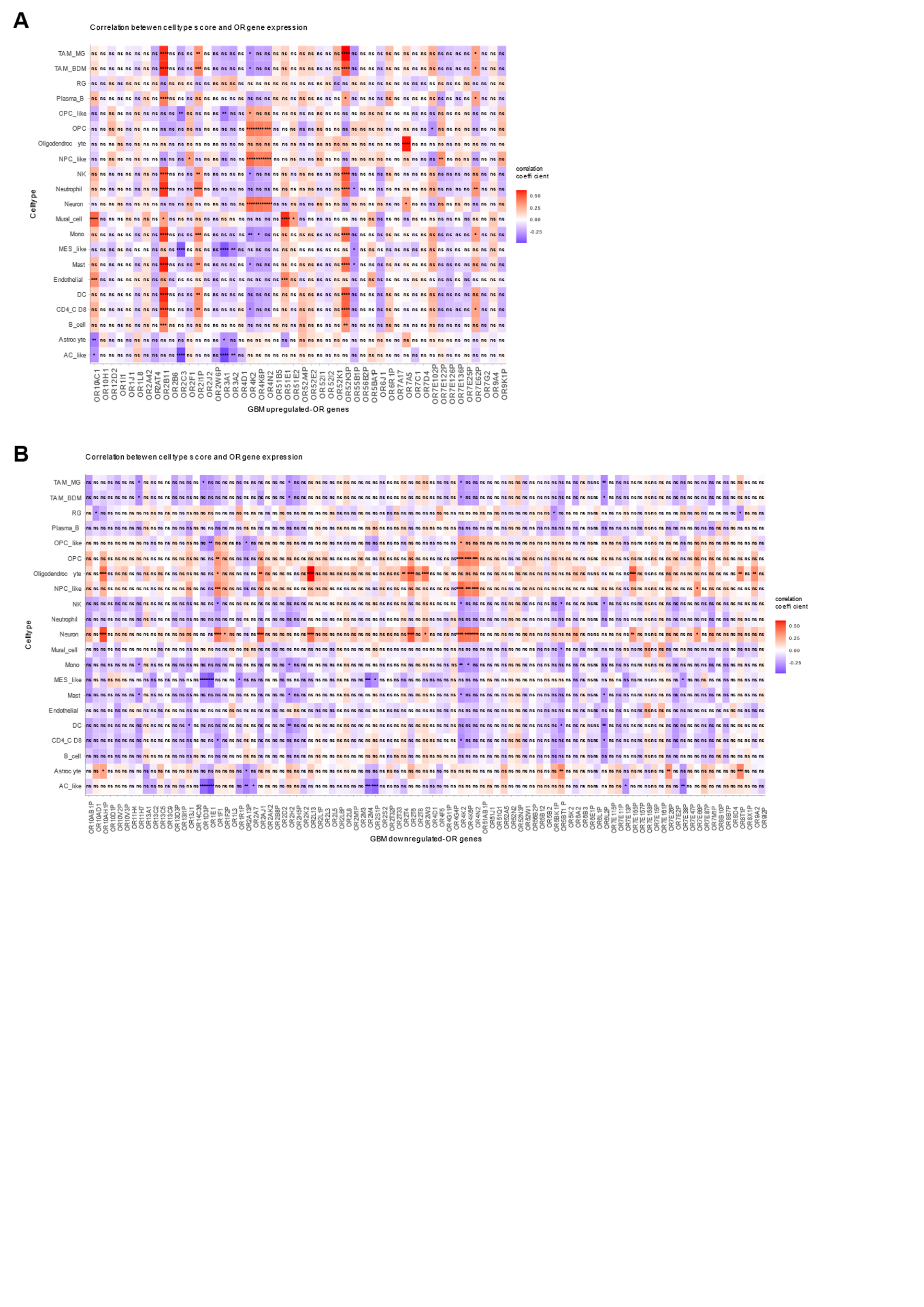


**Figure S2**. Cell-type specificity validation of differentially expressed ORs using TCGA GBM data (**A-B**) Correlation analysis between OR expression levels and cell-type-specific gene signatures in TCGA GBM tissues. The analysis shows correlation patterns for up-regulated (**A**) and down-regulated (**B**) OR genes. Color intensity indicates the Pearson correlation coefficient, with statistical significance assessed by Pearson correlation analysis and adjusted by the Bonferroni method. **** p < 0.0001; *** p < 0.001; ** p < 0.01; * p < 0.05; ns: not significant.


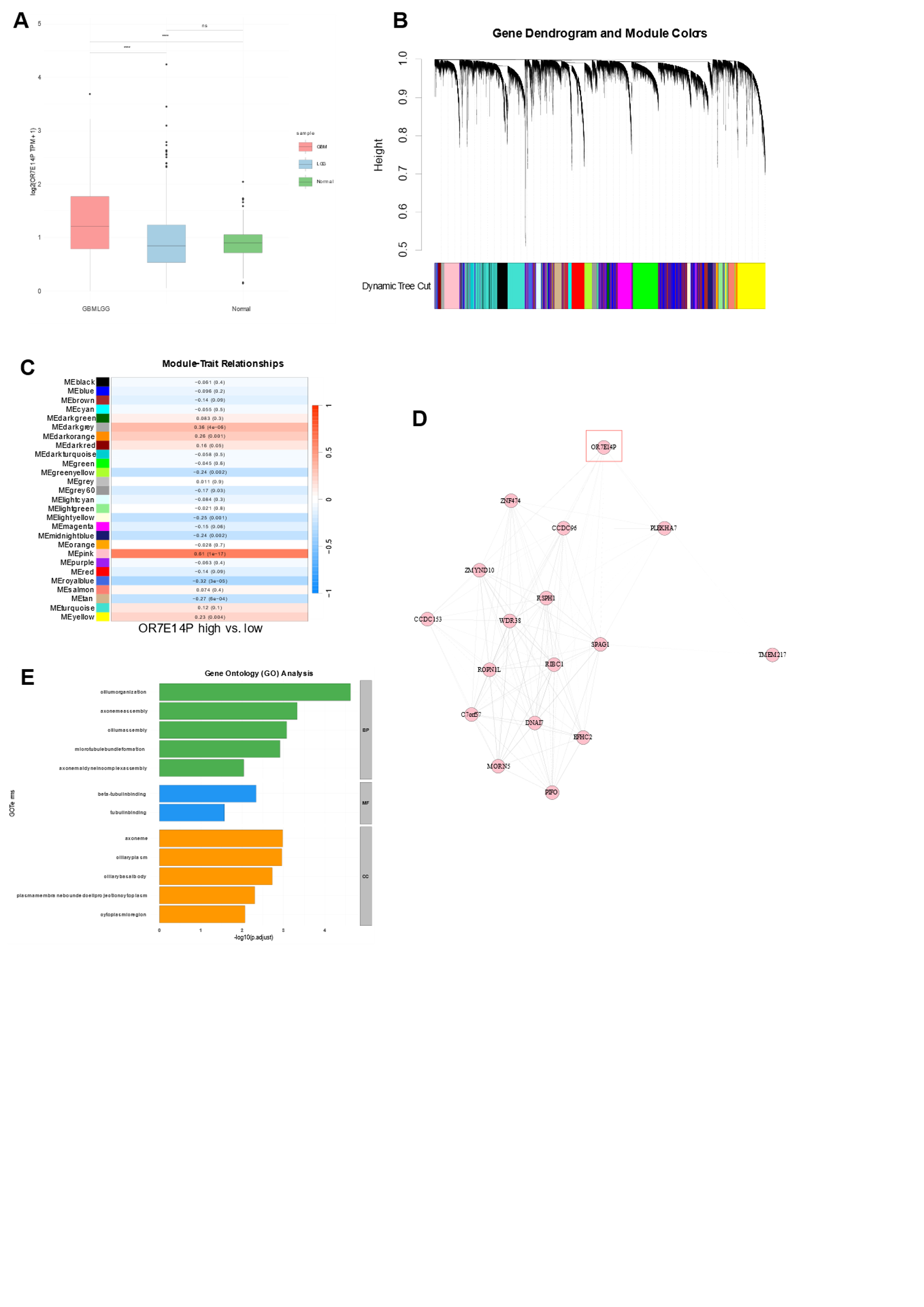


**Figure S3**. Co-expression network analysis of OR7E14P. (**A**) Boxplot showing up-regulated expression of *OR7E14P* across GBM, LGG, and normal brain, with statistical significance determined by Student’s t-test. **** p < 0.0001; *** p < 0.001; ** p < 0.01; * p < 0.05; ns: not significant. (**B**) Gene dendrogram showing the 27 modules identified by WGCNA. (**C**) Module-trait relationship plot for the 27 modules. The trait represents the high *OR7E14P* expression group. Gene significance for each module is shown, with p-values in parentheses. (**D**) Co-expression network showing *OR7E14P* and its highly connected 16 neighbor genes. The red box indicates the *OR7E14P* gene. (**E**) GO results for the genes on the network (D).


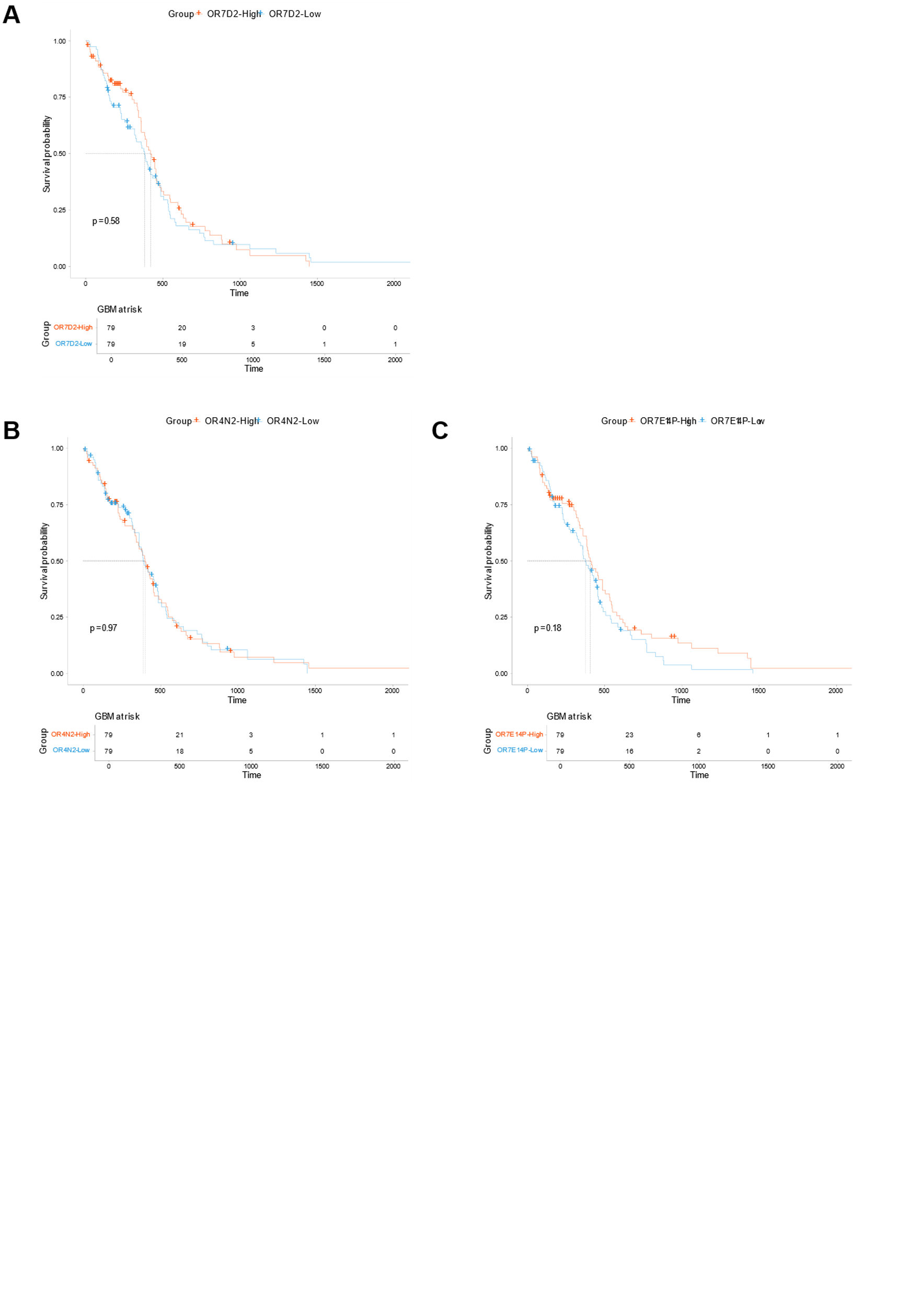


**Figure S4**. Survival analysis of neoplastic cell-enriched ORs in the TCGA GBM cohort (**A-C**) Kaplan–Meier survival analysis comparing high and low expression groups of neoplastic cell-enriched OR genes: *OR7D2* (A), *OR4N2* (B), and *OR7E14P* (C). Statistical significance was assessed by the log-rank test.


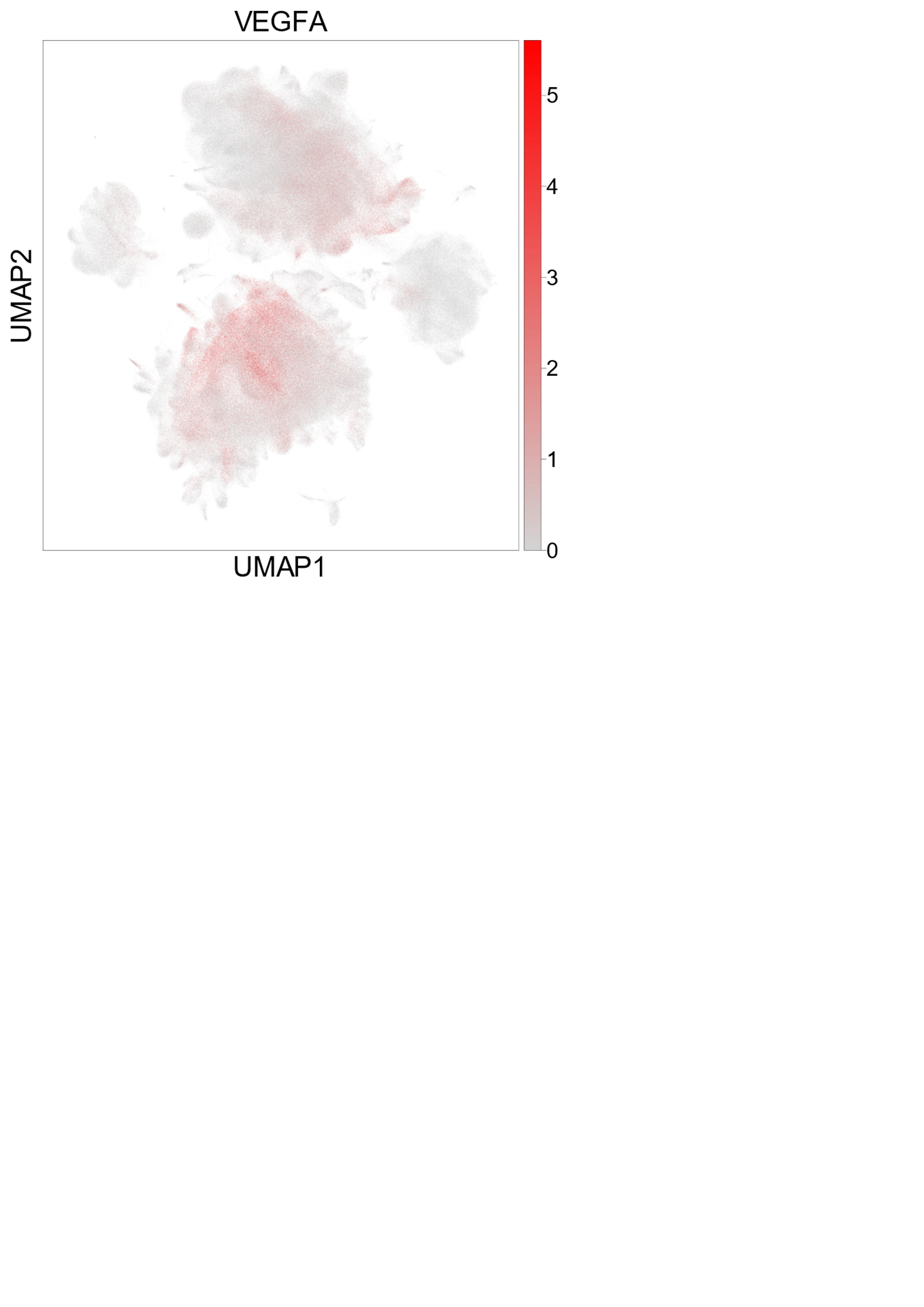


**Figure S5**. Expression distribution of *VEGFA* in GBM single-cell RNA-seq data. UMAP visualization demonstrating *VEGFA* expression patterns across neoplastic and non-neoplastic cell populations, showing predominant expression in neoplastic cells.

**
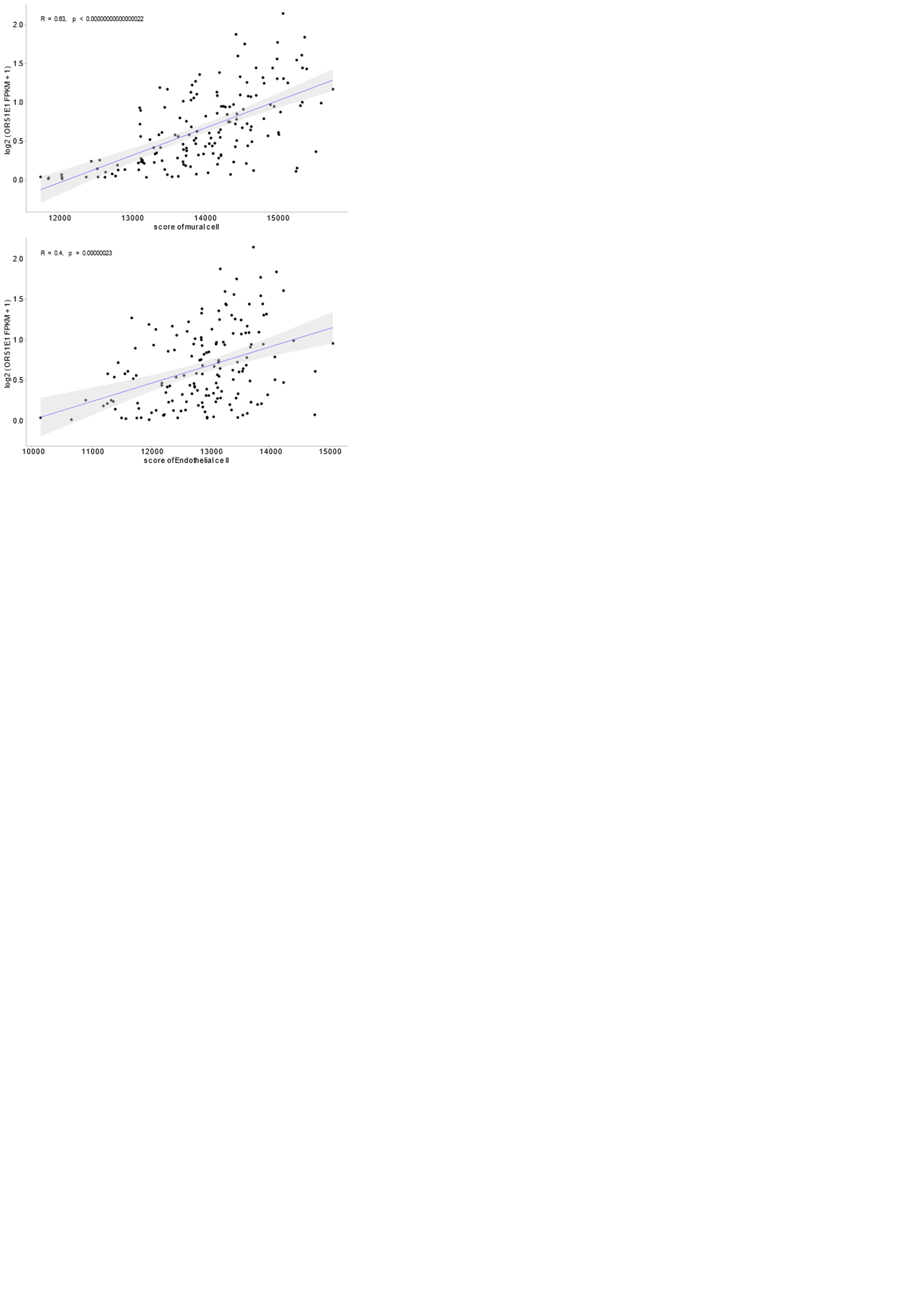
**

**Figure S6**. Correlation analysis of *OR51E1* expression with vascular cell gene sets in TCGA GBM data. Scatter plots showing correlation between *OR51E1* expression and cell-type-specific gene sets. Cell-type scores for mural cells (top) and endothelial cells (bottom) were calculated using ssGSEA. Statistical significance was assessed by Pearson correlation analysis. The solid line represents the linear regression fit with 95% confidence intervals.


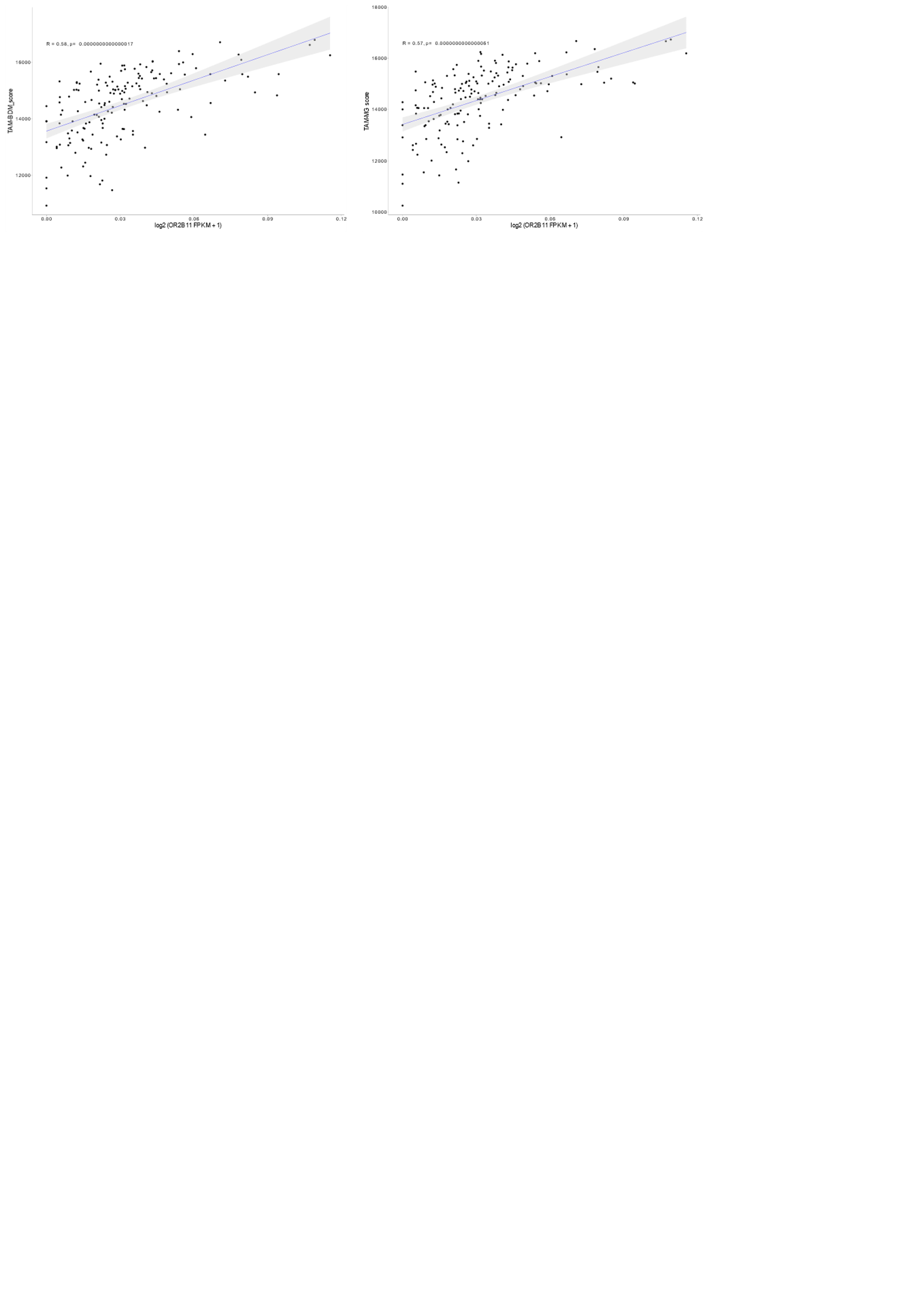


**Figure S7**. Correlation analysis of *OR2B11* expression with TAM gene sets in TCGA GBM data. Scatter plots showing correlation between *OR2B11* expression and cell-type-specific gene sets. Cell-type scores for TAM-BDM (left) and TAM-MG (right) were calculated using ssGSEA. Statistical significance was assessed by Pearson correlation analysis. The solid line represents the linear regression fit with 95% confidence intervals.


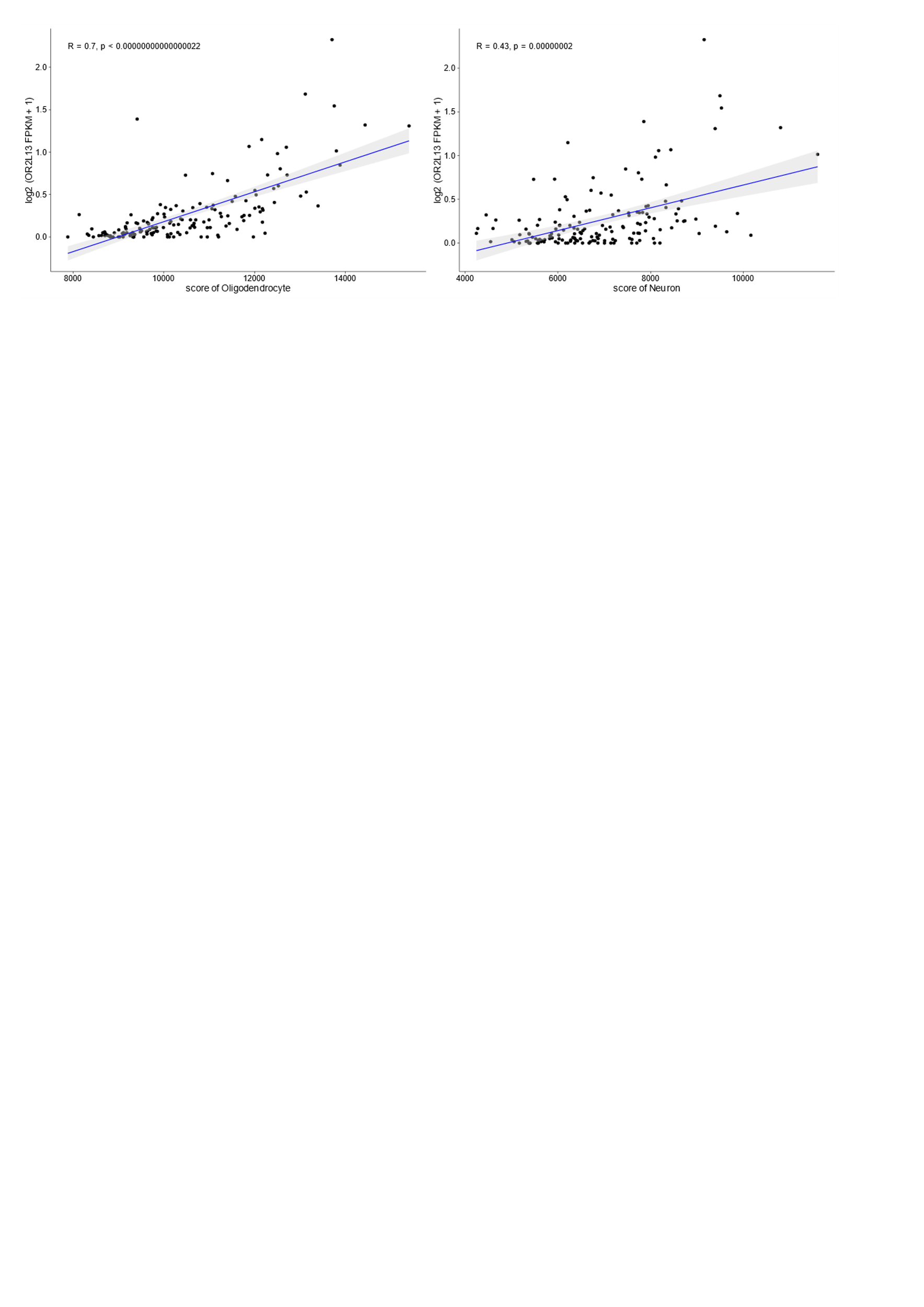


**Figure S8**. Correlation analysis of *OR2L13* expression with oligodendrocyte and neuron gene sets in TCGA GBM data. Scatter plots showing correlation between *OR2L13* expression and cell-type-specific gene sets. Cell-type scores for oligodendrocyte (left) and neuron (right) were calculated using ssGSEA. Statistical significance was assessed by Pearson correlation analysis. The solid line represents the linear regression fit with 95% confidence intervals.
